# Nucleostemin as a possible progenitor marker of corneal epithelial cells

**Published:** 2009-06-10

**Authors:** Motoko Kawashima, Tetsuya Kawakita, Satoru Yoshida, Shigeto Shimmura, Kazuo Tsubota

**Affiliations:** Department of Ophthalmology, Keio University School of Medicine, Tokyo, Japan

## Abstract

**Purpose:**

Nucleostemin, a nuclear protein involved in the regulation of cell cycle and proliferation, is a candidate marker for various stem cells. We examined the expression of nucleostemin as a marker of differentiation and senescence in corneal epithelial progenitor cells.

**Methods:**

Nucleostemin expression in normal mouse corneal epithelium was examined by RT-PCR, as well as in cultured mouse corneal epithelial cells by immunohistochemistry. Co-expression with the progenitor markers p63 and Bmi-1, cytokeratin 14 and 19, the proliferating marker Ki67, differentiation markers cytokeratin 12 and involucrin, and with the senescent marker SA-β-gal were examined by immunohistochemistry. Correlation of cellular size and nucleostemin expression was also examined.

**Results:**

Nucleostemin expression was detected in mouse corneal epithelium by RT-PCR. Immunohistochemistry revealed that nucleostemin was expressed predominantly in basal and suprabasal cells of the whole cornea. The expression of nucleostemin was not associated with the expression of Ki67, K14, and K19, but with the expression of Bmi-1 and particularly with p63. Nucleostemin was not co-expressed with SA-β-gal in same cell.

**Conclusions:**

Nucleostemin can be used as a progenitor marker analogous to p63.

## Introduction

Nucleostemin (guanine nucleotide binding protein-like 3, Gnl3) encodes a nucleolar GTP-binding protein highly enriched in stem cells and cancer cells [1-5], including embryonic stem cells [1,2], neural stem cells [1,2,4], and cancer stem cells [1,5], but not in most terminally differentiated cells [6]. Nucleostemin is thought to be involved in regulation of the cell cycle and proliferation [7]. When nucleostemin expression was knocked down in vitro by siRNA, DNA synthesis failed to pass through the S phase in Hela cells [7] and rat primary bone marrow stromal cells [3,8]. When cell proliferation was stimulated by FGF2, nucleostemin expression was increased in a dose-dependent manner [3], suggesting nucleosteminn expression could be promoted by specific microenvironment. However such an increase of nucleostemin expression by RT-PCR could simply be the result of the proliferation of nucleostemin-positive cells, and not the result of promoting nucleostemin expression within each cell.

In vivo, the embryogenesis of homozygous nucleostemin-null mice was aborted before the blastula stage. Although the growth and fertility of heterozygous nucleostemin-null mice appeared normal, mouse embryonic fibroblasts (MEFs) derived from these mice had less nucleostemin protein, a lower population growth rate, and higher percentages of senescent cells than their wild-type littermates, which could be rescued by transgenic overexpression of nucleostemin [9].

In this study, we investigated the expression of nucleostemin as a possible marker for corneal progenitor/stem cells. To address the issue, we examined the in vivo localization of nucleostemin in the mouse cornea, and compared expression patterns with known markers for progenitor cells, cell proliferation and differentiation. We further used cultured mouse corneal epithelial cells to demonstrate the relationship of nucleostemin expression with cellular senescence.

## Methods

### Tissue preparation and cell culture

C57BL/6 mice (CLEA Japan Inc, Tokyo, Japan), aged 8-10 weeks, were handled according to the guidelines in the ARVO Statement for the Use of Animals in Ophthalmic and Vision Research. Eye globes were enucleated from the mice with forceps after death, washed profusely in phosphate buffered saline (PBS). For immunostaining, whole eyes were cryopreserved. For primary culture, mouse corneal epithelial cells were isolated and cultured by explant method as described previously [10]. Briefly, corneal buttons, including the limbus, were cut from the mouse eye and extraneous tissue removed including the iris and ciliary body. The button was cut in half and each explant with epithelium side up was plated flat on a 6 well plate, one piece per well. After approximately 5-10 min, to allow for attachment of the explant, serum-free low-Ca2^+^ medium (defined keratinocyte serum-free medium, KSFM; Invitrogen, Carlsbad, CA) containing of 10 ng/ml human recombinant EGF (Invitrogen), 100 ng/ml cholera toxin (LIST Biological Laboratories Inc., Campbell, CA), antibiotics, and growth supplement supplied by the manufacturer was added. The cultures were incubated at 37 °C, under 95% humidity and 5% CO_2_ with the medium changed every 3 to 4 days. After approximately 10 days, the explant was carefully transferred to a new dish and cultured as described above. Cells derived from passaged explants were also used for subcultures. Epithelial cells were subcultured by TrypLE Express® (Invitrogen) at 1:3 splits after small cells reached subconfluence, or if the colonies of small cells started to stratify before reaching subconfluence. The procedure was repeated until passage 4 (P4) cultures. The cultures were incubated at 37 °C, under 95% humidity and 5% CO_2_ with the medium changed every 3 to 4 days.

### Reverse transcription–polymerase chain reaction

Total RNA was extracted from mouse corneal epithelium cells cultured for 7 to 10 days using commercial RNA isolation kit (RNeasy; Qiagen, Valencia, CA), and cDNA was synthesized using a kit (RevaTra Ace; Toyobo, Osaka, Japan). The same amount of cDNA was amplified by PCR (GeneAmp 9700; Applied Bioscience, Inc, (ABI), Foster City, CA) for each primer pairs as follows: *Gnl3* (forward; Mm_Gnl3_1242_Fw 5'-AAG CCC AAA TGT GGA AAG TGC-3', reverse; Mm_Gnl3_1706_Rv 5'-TCA TCC TCT TGA CTC GCT CTA TCC-3', product size,465 bp ), *Gapdh* (forward; Mm_Gapdh_565 Fw 5'-GAC CAC AGT CCA TGC CAT CAC-3', reverse; Mm_Gapdh_1107 Rv 5'-TCC ACC ACC CTG TTG CTG TAG-3', product size 453 bp). PCR products were analyzed by agarose gel electrophoresis.

### Immunostaining

Mouse corneal epithelial cells were cultured in twenty-four well plates (10^4^ cells/well) and fixed with 2% paraformaldehyde (PFA, Wako, TX) for the immunostaining of p63, Ki67, Bmi-1, involcrin, K12, K14, and nucleostemin. Corneal tissues were also fixed with 2% PFA for the immunostaining of p63, K12, K14, K19, and nucleostemin. PFA-fixed cells were permeabilized with 0.1% Triton X-100 (Sigma-Aldrich, St. Louis, MO). After background staining was blocked with 10% normal donkey serum, the cells and tissues were treated with the following monoclonal primary antibodies: anti-p63 (4A4; Calbiochem, Darmstadt, Germany), anti-K12 (Transgenic Inc., Kumamoto, Japan), anti-K14 (Abcam, Cambridge, UK), anit-K19 (Neomarker, Fremont, CA), anti-involcrin (SY5; Abcam, Tokyo, Japan), anti-Bmi-1 (1.T.21; Abcam), anti-Ki67 (TEC-3; Dako, Glostrup, Denmark) and anti-nucleostemin (R&D Systems, Minneapolis, MN). The cells or tissues were then treated with Cy3- or FITC-conjugated secondary antibodies (Jackson ImmunoResearch, West Grove, PA). The nuclei were counterstained with 4', 6’-diamino-2-phenylindole (DAPI, 1 μg/ml; Dojindo Laboratories, Tokyo, Japan).

### Analysis of nucleostemin expression

To compare the signal intensity of nucleostemin expression between small and large cells, P2 cells were immunostained against nucleostemin. Staining was scored by two independent observers as ++ (most intense), +, or - (least intense) depending on staining intensity. A minimum of ten fields were examined for each set of 3 wells. Cells were divided into two group according to cell size determined by whether half the length of the major and minor axis was greater or smaller than 100 μm.

### Senescence-associated β-gal staining

Mouse corneal epithelial cells were cultured in 4 well chamber slides (10^4^ cells/well), and fixed after 7 days to detect senescence-associated β-gal staining (SA-β-gal) with a Senescence Detection Kit (BioVision, Mountain View, CA). For double staining, nucleostemin antibody binding was visualized by the avidin-biotin-immuno peroxidase complex method using the Vectastain *Elite* ABC Goat IgG Kit (Vector Laboratories, Inc., Burlingame, CA) according to the manufacturer’s instructions.

## Results

### Nucleostemin expression in mouse cornea in vivo

**Figure 1:**
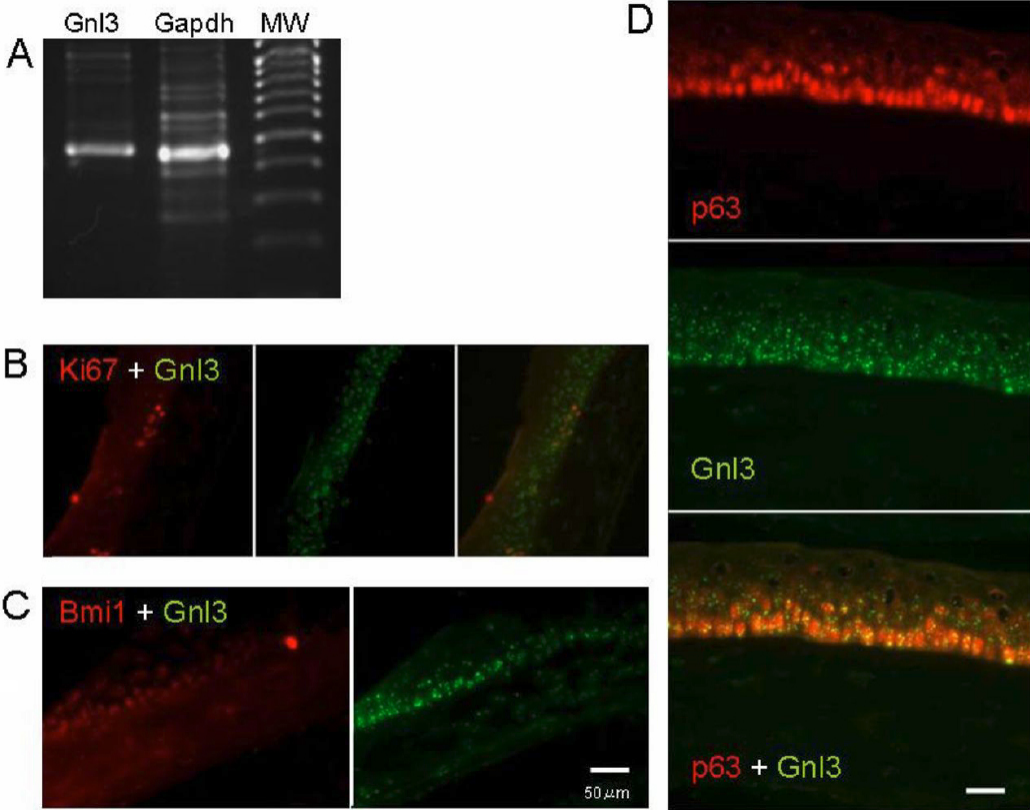
Nucleostemin expression in mouse cornea in vivo. **A**: The nucleostemin (*Gnl3*) band was detected in corneal epithelium by RT-PCR. **B**, **C**, and **D**: Immunostaining of mouse corneal section revealed that nucleostemin was expressed in the basal and suprabasal layer of cornea. Double immunostaining with Ki67 (**B**), Bmi-1(**C**), and p63 (**D**) revealed that p63 was the most similar expression pattern as nucleostemin in vivo mouse cornea. In **C** and **D**, the bars indicate 50 μm.

**Figure 2:**
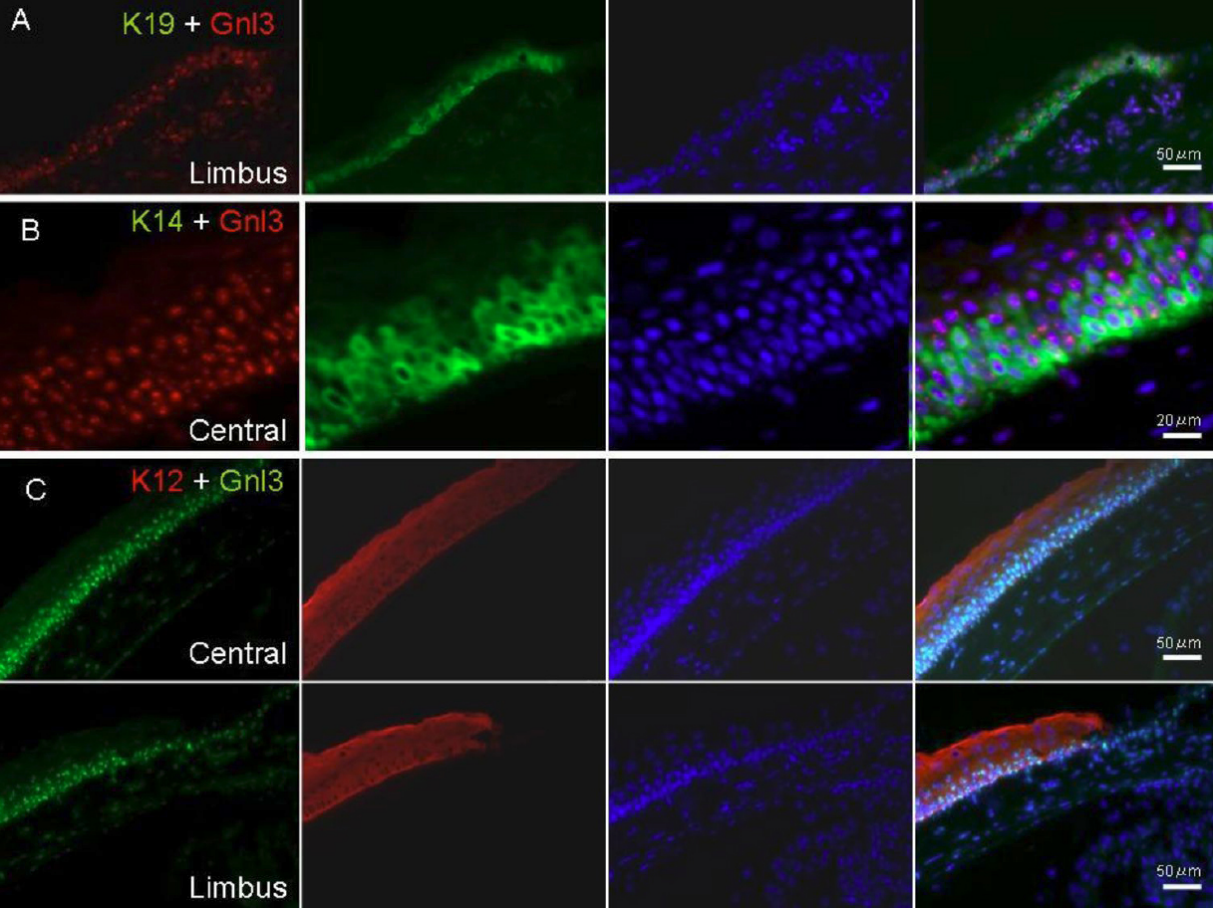
Expression pattern of nucleostemin with cytokeratin in mouse corneal epithelium in vivo. Immunostaining against nucleostemin with cytokeratin 19 (K19), K14, and K12 was performed in mouse corneal epithelium was performed. **A**: K19 was expressed in basal limbal epithelial layer, but nucleostemin was expressed in basal layer of cornea and linmbus. **B**: K14 expression was observed in suparabasal- and basal layer as nucleostemin, but nucleostemin expression was deteced in more cells than K14. K12 was observed in whole corneal layers in central and limbal location (**C**), and nucleostemin expression was similarly detected in central cornea and limbus. The bars indicate 20 (**B**) or 50 μm (**A** and **C**).

In the mouse cornea, a single nucleostemin band was clearly detected by RT-PCR (Figure 1A). In mouse corneal radial sections, immunostaining revealed that nucleostemin was expressed in the basal and suprabasal layers of the corneal epithelia, including the limbus (Figure 1B-D). To further characterize nucleostemin-positive cells, the expression of the proliferation marker Ki67, the progenitor markers p63 and Bmi-1, as well as cytokeratin 12 (K12), K14, and K19 were examined. Double immunostaining with Ki67, Bmi-1, and p63 revealed that p63 had the most similar expression pattern as nucleostemin in vivo (Figure 1B-D). Expression of nucleostemin was compared with K19, K14, and K12 in the mouse corneal epithelium. K19 was expressed in basal layer of limbal epithelium, however, nucleostemin was expressed in the basal layer of both cornea and limbus (Figure 2A). Nucleostemin overlapped with K14 expression in the suparabasal and basal epithelial cells (Figure 2B). K12 was observed in all layers of the central cornea and limbus (Figure 2C).

### Expression of nucleostemin in primary mouse corneal epithelial cultured cells

**Figure 3:**
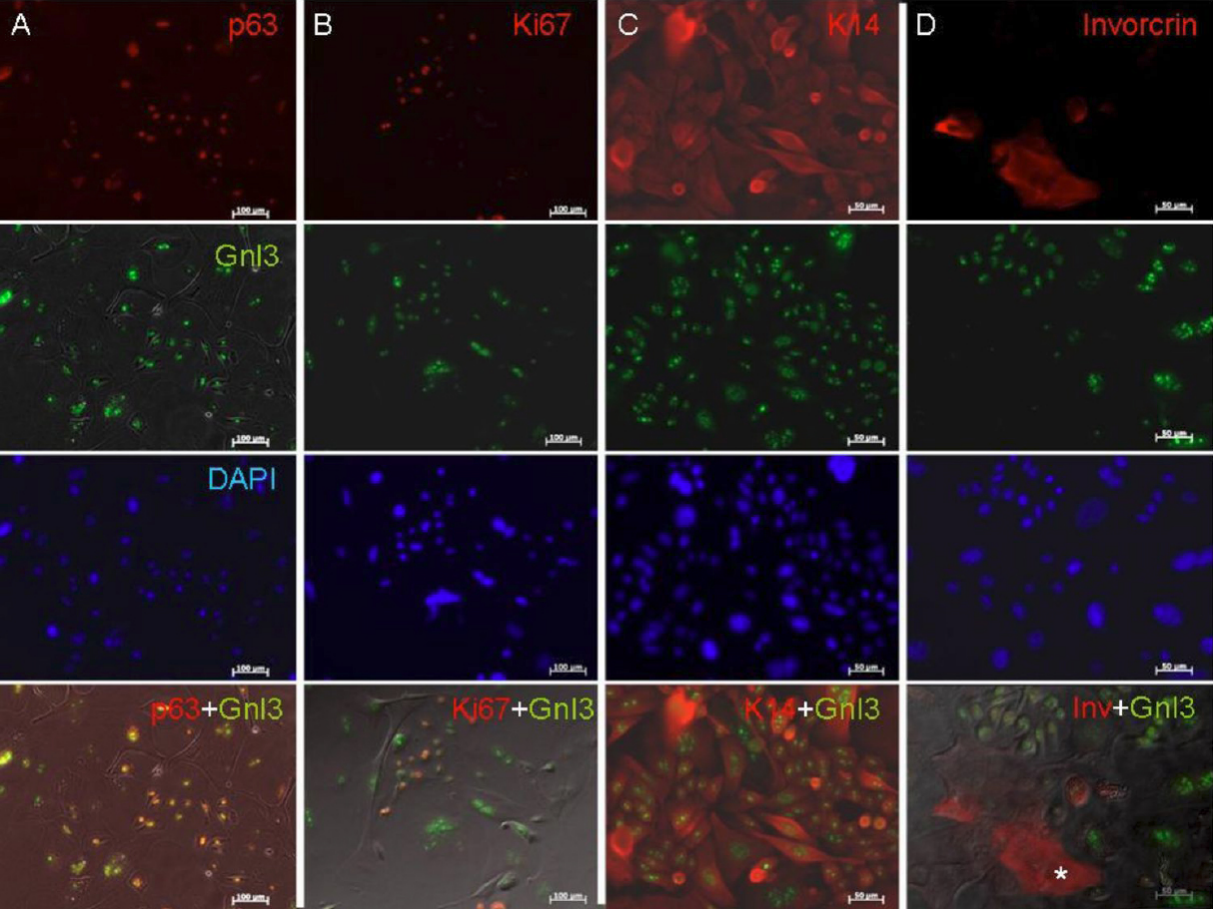
Expression of nucleostemin in primary mouse corneal epithelial culture. Immunostaining against nucleostemin with progenitor (p63), proliferative (Ki67), basal cell (K14), and differentiation marker (involucrin) was performed in primary mouse corneal epithelial culture. The result showed that p63 was mostly overlapped with nucleostemin expression. Ki67 was only expressed in small population of nucleostemin positive cells with small nucleus. K14 was expressed in most of the attached cells accompanied with nucleostemin expression, but some cell showed K14 positive but nucleostemin negative. (indicated by the asteriak) Involucrin was not expressed in nuclestemin positive cells completely. The bars indicate 100μm.

Primary culture of mouse corneal epithelial cells was used for further characterization in vitro. Immunostaining was done against nucleostemin and compared with p63, Ki67, K14, and with the differentiation marker, involucrin. (Figure 3A-D). The result showed that p63 overlapped with nucleostemin, similar to what was observed in the mouse corneal epithelium. The proliferation marker Ki67 was only expressed in a small population of nucleostemin-positive cells with small nuclei. Most of the attached cells expressed K14 and nucleostemin, however, some cell were K14-positive but nucleostemin-negative. Involucrin was exclusively expressed in nuclestemin-negative cells (Figure 3D, indicated by the asterisk).

### Nucleostemin expression and cellular senescence in primary mouse corneal epithelial culture

**Figure 4:**
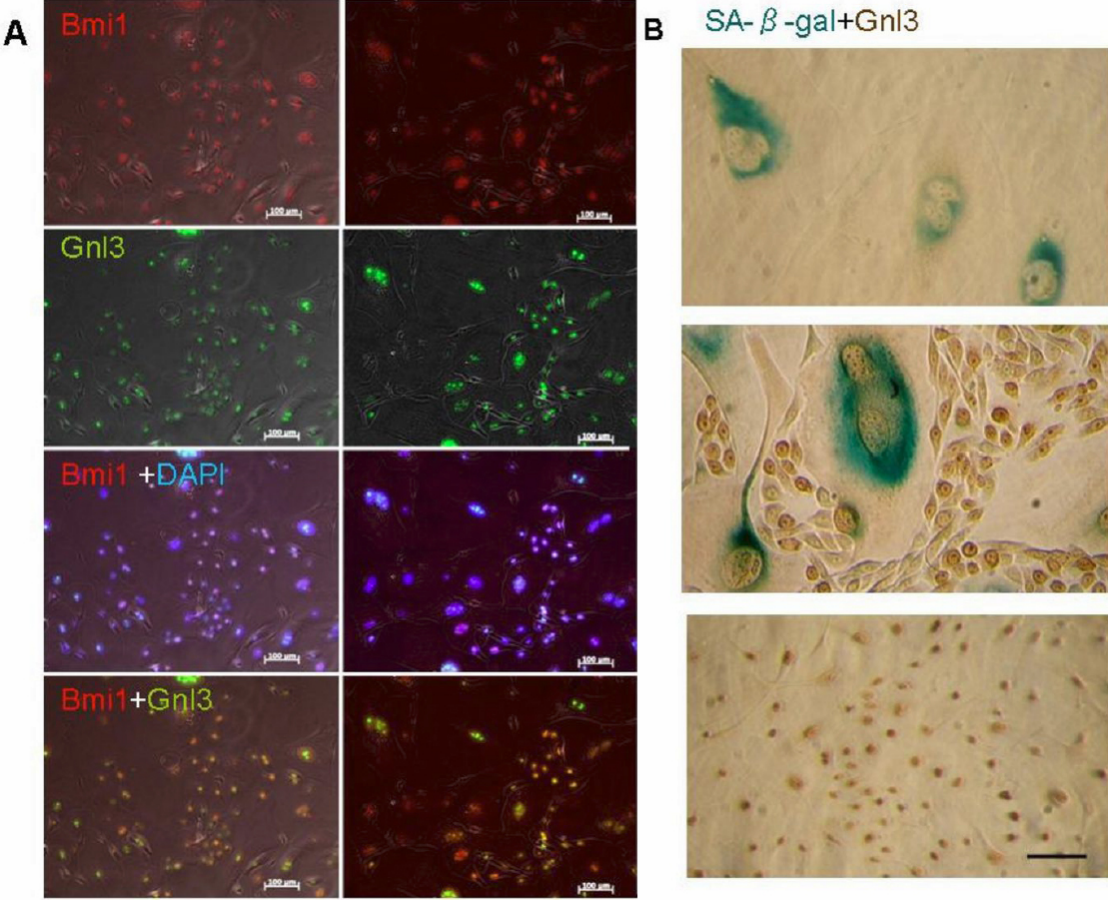
Nucleostemin expression and cellular senescence in primary mouse corneal epithelial culture. Bmi1 expression was mostly overlapped with nucleostemin expression (**A**) as p63 in different location. It was noted that some large cells were also Bmi1-positive and nucleostemin-negative. **A**: Senescent cells display the increase of cell size, and the expression of senescence-associated Beta-galactosidase (SA-ß-gal) activity. Nucleostemin-positive cells were not stained by SA-ß-gal. **B**: The SA-ß-gal is not found in small compact cells (**B**). Bars indicate 100μm.

The expression of Bmi1 decreased with cellular senescence, and was therefore used as a negative marker of cellular senescence. Although nuclear expression of Bmi1 mostly overlapped with nucleostemin in small cells, some of Bmi1-positive large cells were nucleostemin-negative (Figure 4A). SA-ß-gal staining was observed in cells with relatively large cytoplasm and nuclei, while nucleostemin-positive cells and/or small compact cells were completely negative for SA-β- gal staining (Figure 4B).

### Nucleostemin expression and cell size in primary culture

**Figure 5:**
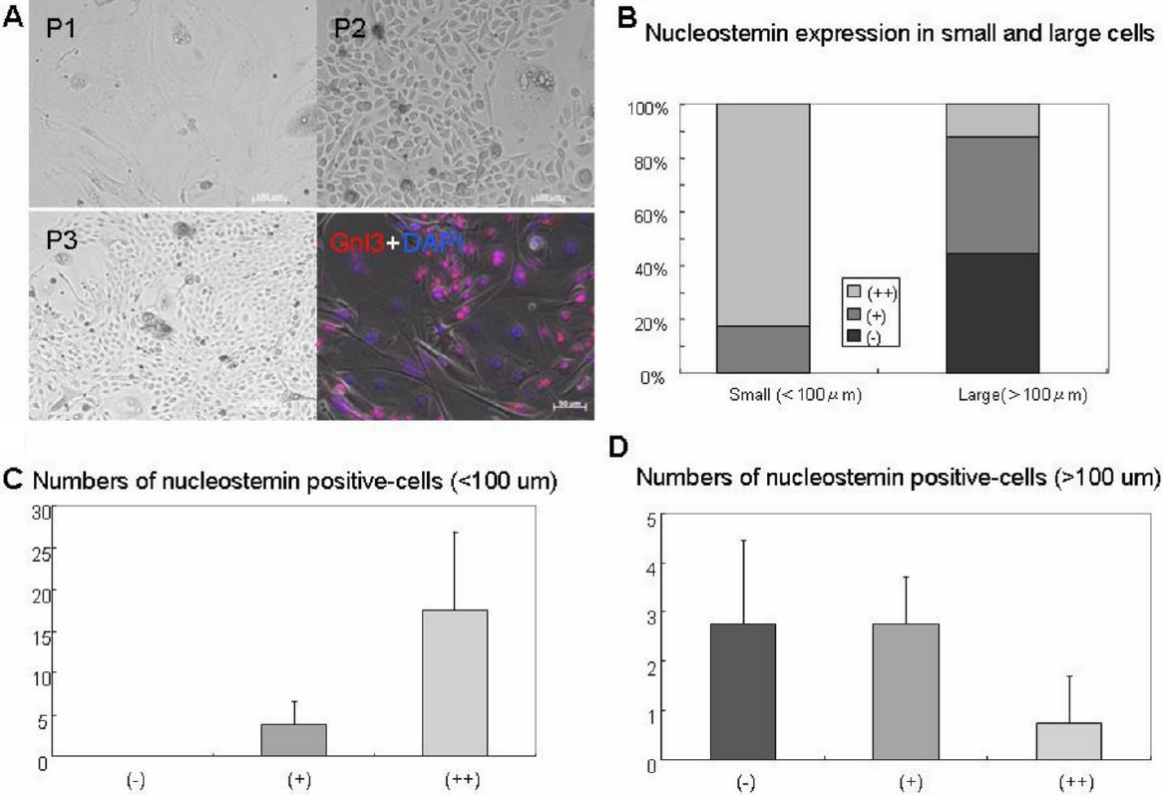
Nucleostemin expression and cell size in primary culture. Cell size was different in early passage (P1-P3) of primary mouse corneal culture, therefore those cells were used for analyzing correlation between cell size and nucleostemin expression. **A**: Immunostaining in P2 showed that small cells were strongly positive in nucleostemin, but negative in large cells. All of the cells with the cell size of smaller than 100 μm expressed nucleostemin (**B** and **C**), however cells with the size of larger than 100 μm showed less or no expression of nucleostemin (**B** and **D**).

Cell size varied during early passages (P1-P3) of primary mouse corneal epithelial culture, and therefore these cells were used for analyzing the correlation between cell size and nucleostemin expression (Figure 5A). Immunostaining in P2 showed that small cells were strongly positive for nucleostemin, but large cells were negative. Signal intensity of nucleostemin was significantly higher in small cells compared to large cells (Figure 5A). All cells with a cell size of smaller than 100 μm expressed nucleostemin, while cells lager than 100 μm showed little or no expression of nucleostemin (Figure 5B,C,D).

## Discussion

The expression of nucleostemin has been reported in proliferating cells in various tissues including bone marrow stromal cells [1-3], malignancies [1,5,11,12], and skin [13]. In skin, nuclostemin was expressed more in keratinocyte on feeder cells than those without feeder cells [13], which supported the fact that nucleostemin may be used as a progenitor marker for stratified epithelial cells. However, corneal epithelial cells are different from epidermal keratinocytes due to its avascularity, and a non-keratinized, transparent morphology. At present, no data concerning its expression in normal corneal epithelia are available. In this study, nucleostemin expression was observed in vivo and in vitro mouse corneal epithelial cells. Although the immunostaining pattern in corneal epithelium was similar to epidermal tissue, no staining was observed in corneal stroma, unlike in dermal tissue. This discrepancy could be explained by the fact that cell mitosis is more frequent in epidermis than corneal stroma.

Comparing nucleostemin expression with various markers showed that p63 (4A4) showed the most similar staining pattern in the mouse cornea both in vivo and in vitro. The expression of p63 is often used as a progenitor marker for keratinocytes and cornea [14,15]. The localization of p63 by immunostaining was observed as uniform staining of the nucleus, which is sometimes confused with non-specific background staining. However the expression of nucleostemin was observed as strong signals from the nucleoli, which is much more specific compared to p63, and therefore, may be superior as a progenitor marker. Bmi1, recognized as a progenitor marker for cornea, revealed similar expression with nucleostemin in vitro. However, in vivo, nucleostemin expression was broader than Bmi1, which was reported to decrease during aging and cellular senescence [16,17].We used subcultured cells for this experiments, which could explain the difference between in vivo and in vitro.

Nucleostemin expression is an indicator of the proliferative capacity of cells. On the other hand, nucleostemin expression is not directly connected to proliferation. Since nucleostemin is widely expressed in cells of normal and malignant renal tissue [11], it is reasonable to presume that nucleostemin has tasks other than simply regulating proliferation. In primary rat cortical stem cells, nucleostemin expression appears to control proliferation and onset of apoptosis. The lack of nucleostemin was connected with the lack of cell proliferative activity, but the overexpression of nucleostemin was connected with the induction of apoptosis [1]. In this study, all Ki67-positive cells were strongly positive for nucleostemin, and many Ki67-negative cells also maintained nucleostemin expression in the corneal basal and suprabasal epithelial layers. The strength of nucleostemin expression in the cornea may not correlate with the induction of apoptosis in vivo.

K14 is expressed in basal epithelial cells with the capacity for stratification [18,19], and nucleostemin expression was similar to K14 in vivo. In vitro, K14 was expressed in most attached cells, while nucleostemin was expressed in a subset of K14-positive cells, indicating that K14 expression may not be related to the proliferating capacity of cells.

By analyzing the correlation between cell size and nucleostemin expression, the decrease or absence of nucleostemin was observed in large flatten cells. Immunostaining showed that small cells were strongly positive in nucleostemin, but negative in large cells. Furthermore, SA-β-gal positive cells did not express nucleostemin, which shows that nucleostemin is not expressed in senescent cells.

In conclusion, nucleostemin is available as a marker for p63-positive progenitor and non-senescent cells of the corneal epithelium. The unique staining pattern of nucleostemin within the nucleoli may have an advantage over p63, in which background staining and isotype specificity is often debated.
